# Cardiac-Specific Overexpression of Metallothionein Rescues against Cigarette Smoking Exposure-Induced Myocardial Contractile and Mitochondrial Damage

**DOI:** 10.1371/journal.pone.0057151

**Published:** 2013-02-19

**Authors:** Nan Hu, Xuefeng Han, Erin K. Lane, Feng Gao, Yingmei Zhang, Jun Ren

**Affiliations:** 1 Center for Cardiovascular Research and Alternative Medicine, University of Wyoming College of Health Sciences, Laramie, Wyoming, United States of America; 2 Department of Physiology, Xijing Hospital, Fourth Military Medical University, Xi'an, China; 3 Department of Cardiology, Xijing Hospital, Fourth Military Medical University, Xi'an, China; Virginia Commonwealth University Medical Center, United States of America

## Abstract

**Objectives:**

Second hand cigarette smoke is an independent risk factor for cardiovascular disease. Although a tie between smoking and cardiovascular disease is well established, the underlying mechanisms still remains elusive due to the lack of adequate animal models. This study was designed to use a mouse model of exposure to cigarette smoke, a surrogate of environmental tobacco smoke, to evaluate the impact of cardiac overexpression of heavy metal scavenger metallothionein on myocardial geometry, contractile and intracellular Ca^2+^ properties and apoptosis following side-stream smoke exposure.

**Methods:**

Adult male wild-type FVB and metallothionein transgenic mice were placed in a chamber exposed to cigarette smoke for 1 hour daily for 40 days. Echocardiographic, cardiomyocyte contractile and intracellular Ca^2+^ properties, fibrosis, apoptosis and mitochondrial damage were examined.

**Results:**

Our data revealed that smoke exposure enlarged ventricular end systolic and diastolic diameters, reduced myocardial and cardiomyocyte contractile function, disrupted intracellular Ca^2+^ homeostasis, facilitated fibrosis, apoptosis and mitochondrial damage (cytochrome C release and aconitase activity), the effects of which were attenuated or mitigated by metallothionein. In addition, side-stream smoke expose enhanced phosphorylation of Akt and GSK3β without affecting pan protein expression in the heart, the effect of which was abolished or ameliorated by metallothionein. Cigarette smoke extract interrupted cardiomyocyte contractile function and intracellular Ca^2+^ properties, the effect of which was mitigated by wortmannin and NAC.

**Conclusions:**

These data suggest that side-stream smoke exposure led to myocardial dysfunction, intracellular Ca^2+^ mishandling, apoptosis, fibrosis and mitochondrial damage, indicating the therapeutic potential of antioxidant against in second smoking-induced cardiac defects possibly via mitochondrial damage and apoptosis.

## Introduction

Chronic cigarette smoking predisposes individuals to various chronic diseases including cardiovascular diseases through overtly increased systemic oxidative stress 1]. Epidemiologic survey has indicated that cigarette smoking may increase the incidence of myocardial infarction and fatal coronary artery disease 2]. Even low-tar exposure of cigarette is known to drastically enhance the prevalence of cardiovascular diseases compared with non-smokers 2,3]. In addition, passive smoking (second-hand smoking) may be associated with a 30% increase in the risk of coronary artery diseases compared with an 80% increase in activate smokers 2,4]. Given that smoking is a preventable global problem, a better understanding of how cigarette and second-hand smoking contribute to the pathogenesis of cardiovascular diseases is pivotal to the proper management of smoking-related premature death 2,4]. One of the main challenges to examine the pathogenesis of smoking-induced cardiovascular disorders is the lack of appropriate animal models. While mice are widely employed in experimental medicine (such as smoking-induced lung diseases) and can offer a unique potential for genetic manipulation 5], few studies were performed with regards to the underlying mechanisms of cigarette smoking on cardiac function.

Cigarette smoking is associated with cardiac remodeling and accumulation of oxidative stress 1,6,7]. Recent evidence has suggested a beneficial role of antioxidants against ischemia-induced neovascularization in mice exposed to cigarette smoke 8]. To this end, this study was designed to examine the effect of cardiac-specific overexpression of the heavy metal scavenging antioxidant metallothionein on side-stream smoke exposure, which mimics second hand smoking, induced myocardial contractile dysfunction, if any. Metallothionein is capable of displacing free radicals and regulating redox and apoptotic states and has been shown to benefit a number of cardiovascular anomies in drug-induced cardiac toxicity, aging, sepsis, obesity and diabetes 9]. One recent study from our laboratory depicted that metallothionein protected against *in vivo* nicotine exposure-induced cardiac anomalies via alleviation of reactive oxygen species (ROS) accumulation and apoptosis 10]. In an effort to understand the mechanism of action behind side-stream smoke exposure and metallothionein-induced myocardial responses, intracellular Ca^2+^ handling, fibrosis, apoptosis and mitochondrial function damage were assessed in the hearts from friendly virus B (FVB) wild-type and cardiac-specific metallothionein overexpression transgenic mice. Given that ROS production, apoptosis and mitochondrial function are closely associated with phosphorylation of Akt, an essential cardiac survival factor, and its downstream signal glycogen synthase kinase 3β (GSK3β) in the heart 11], basal activation of Akt and GSK3β was also scrutinized in hearts from FVB and metallothionein mice following side-stream smoke exposure.

## Materials and Methods

### Experimental animals and Side-stream smoke exposure

The animal procedures described in this study were approved by the University of Wyoming Institutional Animal Use and Care Committee (Laramie, WY). In brief, adult male mice with a ten-fold cardiac-specific transgenic overexpression of the heavy metal scavenger metallothionein driven by the mouse α-MHC promoter were generated from the albino friend virus B (FVB) mice as described previously 9]. FVB mice were employed as wild-type mice. FVB and metallothionein mice were placed in an exposure box with 1 cigarette smoke for 1 hour daily for 40 days as described (Golden Monkey; TAR: 13 mg; Nicotine: 1.1 mg; CO: 15 mg) 12]. The cigarette smoke concentration used in our study was 125.5±8.4 mg/m^3^ (n = 8) total suspended particulates (TSP), similar to the levels reported previously 13]. Body weight and blood pressure were assessed regularly with a laboratory scale and a semiautomated tail cuff device (IITC, Woodland Hills, CA). Non-invasive echocardiographic assessment, blood pressure and terminal tissue collection were conducted within 2 hrs after the final side-stream smoke exposure. All mice were maintained at 22°C with a 12/12-light/dark cycle and received lab chow and water *ad libitum*.

### Plasma analysis

To evaluate the effect of side-stream smoke exposure and cardiac-specific overexpression of metallothionein on systemic metabolism, inflammation and oxidative stress, plasma levels of glucose, insulin, tumor necrosis factor-α (TNF-α), interleukin 6 (IL-6), and free-8-isoprostane were measured using commercially available glucometer (Accu-ChekII, model 792, Boehringer Mannheim Diagnostics, Indianapolis, IN) or immunoassay kits (Diagnostic System Laboratory, Webster, TX, Millipore, Billerica, MA and Cayman Chemical, Ann Arbor, MI) 14,15]. Plasma levels of nitric oxide (NOx including NO_2_^−^ and NO_3_^−^) were determined colorimetrically after mixing 100 µl plasma and Griess reagent [0.1% N-(1-naphthyl) ethylenediamine in water and 1% sulfanilamide in 5% phosphoric acid] 16].

### Reduced and oxidized glutathione (GSH and GSSG)

Myocardial samples were homogenized in 1% picric acid and 5% trichloroacetic acid to prevent GSH autoxidation. Acid homogenates were centrifuged at 16,000× g (30 min) and supernatant fractions were collected for GSH and GSSG assay. Half of each sample was used for GSH determination and the other half for GSSG. Samples for GSSG determination were incubated at room temperature with 2 µl of 4-vinyl pyridine (4-VP) per 100 µl sample for 1 hr after vigorous vortexing. Incubation with 4-VP conjugates any GSH present in the sample so that only GSSG is recycled to GSH in the recycling assay. This allowed for measurement of only GSSG without interference by GSH. The GSSG (as GSHx2) was subtracted from the total glutathione to determine actual GSH level 17].

### Echocardiographic assessment

Cardiac geometry and function were evaluated in anesthetized (ketamine 80 mg/kg and xylazine 12 mg/kg, i.p.) mice using 2-D guided M-mode echocardiography (Sonos 5500) equipped with a 15–6 MHz linear transducer. Left ventricular anterior and posterior wall dimensions during diastole and systole were recorded from three consecutive cycles in M mode using methods adopted by the American Society of Echocardiography. Fractional shortening was calculated from LV end-diastolic (EDD) and end-systolic (ESD) diameters using the equation (EDD−ESD)/EDD. Heart rate was calculated from 20 consecutive cardiac cycles 18].

### Isolation of cardiomyocytes

Murine cardiomyocytes were isolated as described 18]. After ketamine/xylazine sedation, hearts were removed and perfused with Ca^2+^-free Tyrode's solution containing (in mM): NaCl 135, KCl 4.0, MgCl_2_ 1.0, HEPES 10, NaH_2_PO_4_ 0.33, glucose 10, butanedione monoxime 10, and the solution was gassed with 5% CO_2_/95% O_2_. Hearts were digested with Liberase Blendzyme 4 (Hoffmann-La Roche Inc., Indianapolis, IN) for 20 min. Left ventricles were removed and minced before being filtered. Tissue pieces were gently agitated and pellet of cells was resuspended. Extracellular Ca^2+^ was added incrementally back to 1.20 mM over a period of 30 min. Isolated myocytes were used within 8 hrs of isolation. Normally, a yield of 50–60% viable rod-shaped cardiomyocytes with clear sarcomere striations was achieved. Only rod-shaped myocytes with clear edges were selected for mechanical study.

### Cell shortening/relengthening

Mechanical properties of cardiomyocytes were assessed using a SoftEdge MyoCam system (IonOptix, Milton, MA). In brief, cells were placed in a Warner chamber mounted on the stage of an inverted microscope (Olympus IX-70) and superfused (∼1 ml/min at 25°C) with a buffer containing (in mM) 131 NaCl, 4 KCl, 1 CaCl_2_, 1 MgCl_2_, 10 glucose, and 10 HEPES at pH 7.4. The cells were field stimulated with suprathreshold voltage at a frequency of 0.5 Hz using a pair of platinum wires placed on opposite sides of the chamber connected to a FHC stimulator (Brunswick, NE). The myocyte being studied was displayed on the computer monitor using an IonOptix MyoCam camera. An IonOptix SoftEdge software was used to capture changes in cell length during shortening and relengthening. Cell shortening and relengthening were assessed using the following indices: resting cell length, peak shortening (PS), time-to-PS (TPS), time-to-90% relengthening (TR_90_), and maximal velocity of shortening/relengthening (± dL/dt) 18].

### Intracellular Ca^2+^ transients

A cohort of myocytes was loaded with fura-2/AM (0.5 µM) for 10 min, and fluorescence intensity was recorded with a dual-excitation fluorescence photomultiplier system (IonOptix). Myocytes were placed onto an Olympus IX-70 inverted microscope and imaged through a Fluor 40 oil objective. Cells were exposed to light emitted by a 75 W lamp and passed through either a 360 or a 380 nm filter, while being stimulated to contract at 0.5 Hz. Fluorescence emissions were detected between 480 and 520 nm, and qualitative change in fura-2 fluorescence intensity (FFI) was inferred from the FFI ratio at the two wavelengths (360/380). Fluorescence decay time was measured as an indication of the intracellular Ca^2+^ clearing rate. Single exponential curve fit was used to calculate the intracellular Ca^2+^ decay constant 10].

### Histological examination

Following anesthesia, hearts were excised and immediately placed in 10% neutral-buffered formalin at room temperature for 24 hrs after a brief rinse with PBS. The specimen were embedded in paraffin, cut in 5 µm sections and stained with hematoxylin and eosin (H&E). Cardiomyocyte cross-sectional areas were calculated on a digital microscope (x400) using the Image J (version1.34S) software. The Masson's trichrome staining was used to detect fibrosis in heart sections. The percentage of fibrosis was calculated using the histogram function of the Photoshop software. Briefly, 12 random fields at 400× magnification from each section were assessed for fibrosis. The fraction of the light blue stained area normalized to the total area was used as an indicator of myocardial fibrosis while omitting fibrosis from the perivascular, epicardial and endocardial regions 19].

### Generation of intracellular reactive oxygen species (ROS)

To evaluate generation of myocardial ROS, freshly frozen left ventricular myocardium (10 µm slices) was incubated for 1 hr at 37°C with 5-(6)-chloromethyl–2′,7′-dichlorodihydrofluorescein diacetate (4 µM) as described 20]. Cardiac tissues were sampled using an Olympus BX-51 microscope equipped with an Olympus MagnaFire™ SP digital camera and ImagePro image analysis software (Media Cybernetics, Silver Spring, MD). Fluorescence was calibrated with InSpeck microspheres (Molecular Probes).

### Aconitase activity

Mitochondrial aconitase, an iron-sulfur enzyme located in citric acid cycle, is readily damaged by oxidative stress via removal of an iron from [4Fe–4S] cluster. Mitochondrial fractions prepared from whole heart homogenate were resuspended in 0.2 mM sodium citrate. Aconitase activity assay (Aconitase activity assay kit, Aconitase-340 assay^TM^, OxisResearch, Portland, OR) was performed according to manufacturer instructions with minor modifications. Briefly, mitochondrial sample (50 µl) was mixed in a 96-well plate with 50 µl trisodium citrate (substrate) in Tri-HCl pH 7.4, 50 µl isocitrate dehydrogenase (enzyme) in Tris-HCl, and 50 µl NADP in Tris-HCl. After incubating for 15 min at 37°C, the absorbance was dynamically recorded at 340 nm every min for 5 min with a spectrophotometer. During the assay, citrate is isomerized by aconitase into isocitrate and eventually α-ketoglutarate. The Aconitase-340 assay^TM^ measures NADPH formation, a product of the oxidation of isocitrate to α-ketoglutarate. Tris-HCl buffer (pH 7.4) was served as blank 21].

### Separation of mitochondrial and cytosolic fractions

Ventricles were minced and homogenized by Polytron in the ice-cold MSE buffer [220 mM mannitol, 70 mM sucrose, 2 mM EGTA, 5 mM 3-(4-morpholino) propane sulfonic acid (MOPS), pH 7.4, 0.2% bovine serum albumin (BSA) and a protease inhibitor cocktail containing 4-(2-aminoethyl) benzenesulfonyl fluoride (AEBSF), E-64, bestatin, leupeptin, aprotinin, and EDTA obtained from Sigma Chemicals (St. Louis, MO)]. The homogenates were centrifuged for 10 min at 600× g to remove unbroken tissue and nuclei, and the supernatants were centrifuged for 10 min at 3000× g to pellet mitochondria. The supernatants were further centrifuged for 30 min at 100,000× g to obtain cytosolic fraction. The mitochondrial pellet was dissolved in a lysis buffer and centrifuged at 10,000× g for 30 min at 4°C to make a soluble protein. Fifty µg of the mitochondrial or cytosolic protein was separated by 15% sodium dodecyl sulfate polyacrylamide gel electrophoresis (SDS-PAGE) for western blot analysis of cytochrome C 22].

### Western Blot Analysis

Protein samples were prepared as described 18]. Samples containing equal amount of proteins were separated on 10% SDS-polyacrylamide gels in a minigel apparatus (Mini-PROTEAN II, Bio-Rad, Hercules, CA) and transferred to nitrocellulose membranes. The membranes were blocked with 5% milk in TBS-T, and were incubated overnight at 4°C with anti-metallothionein, anti-Bax, anti-Bcl-2, anti-cleaved caspase-3, anti-cleaved caspase-9, anti-cleaved caspase-12, anti-Akt, anti-phospho-Akt, anti-GSK3β, anti-phospho-GSK3β, anti-sarco(endo)plasmic reticulum Ca^2+^-ATPase isozyme 2a (SERCA2a), anti-phospholamban, anti-phospho-phospholamban, anti-eNOS, anti-phospho-eNOS, anti-p53 and anti-cytochrome C antibodies. After washing blots to remove excessive primary antibody binding, blots were incubated for 1 hr with horseradish peroxidase (HRP)–conjugated secondary antibody (1∶5,000). Antibody binding was detected using enhanced chemiluminescence (Amersham Pharmacia, Piscataway, NJ), and film was scanned and the intensity of immunoblot bands was detected with a Bio-Rad Calibrated Densitometer (Model: GS-800). All tissue samples were run in duplicates. GAPDH was used as the loading control.

### Immunoprecipitation of oxidized SERCA2a

Myocardium were homogenized and sonicated in a buffer containing 0.5% CHAPS (3-[(3-cholamidopropyl) dimethylammonio]-1-propanesulfonate; 1 mg CHAPS/100 µg protein), 10 mM Tris–HCl (pH 7.4), 50 mM dithiothreitol, 0.3 M sucrose, with protease inhibitors at 4°C. After centrifugation (6,000×g, 10 min), the anti-SERCA2a antibody (Affinity BioReagent, Denver, CO) was added to the supernatant and was incubated overnight at 4°C. An IgG–agarose slurry was added and rotary-mixed at 4°C for 2 hrs. Oxidized SERCA2a was probed immunochemically after derivatization with dinitrophenylhydrazine. Total SERCA2a expression after immunoprecipitation was quantified and was used to normalize protein loading 23].

### Preparation of the side-stream smoke extract (SSE) and in vitro assessment of cardiomyocyte function and intracellular Ca^2+^ properties

SSE was prepared using a side-stream apparatus as described 24]. Side-stream smoke was collected from 1 filtered commercial cigarette (Golden Monkey; TAR: 13 mg; Nicotine: 1.1 mg; CO: 15 mg) which was smoked for 5 min. During the side-stream extraction, all smoke was collected from the smoldering end of the cigarette, and none passes through the cigarette or filter. The smoke was bubbled into a flask containing 50 ml of HEPES-buffered saline (130 mM NaCl, 20 mM HEPES-NaOH, pH 7.4) to generate a solution of SSE of cigarette smoke (1.8 mg/ml). Extracts were then separated into aliquots and stored at −20°C. To assess the effect of SSE on cardiomyocyte contractile function and the underlying mechanism involved, freshly isolated murine cardiomyocytes from FVB mice were pretreated with the antioxidant N-acetylcysteine (NAC, 500 µM)25], the PI3K inhibitor wortmannin (100 nM) 26] or the GSK3β inhibitor SB216763 (100 nM) 27] for 1 hr prior to exposing to 5% SSE (90 µg/ml) 28,29] for an additional 1 hr. Mechanical function and intracellular Ca^2+^ transients were then evaluated in cardiomyocytes.

### Statistical analysis

Data were mean ± SEM. Statistical significance (p<0.05) was estimated by a two-way analysis of variance (ANOVA) followed by a Bonferroni multi-comparison analysis when necessary.

## Results

### General and echocardiographic properties of FVB and metallothionein mice following side-stream smoke exposure

**Figure 1:**
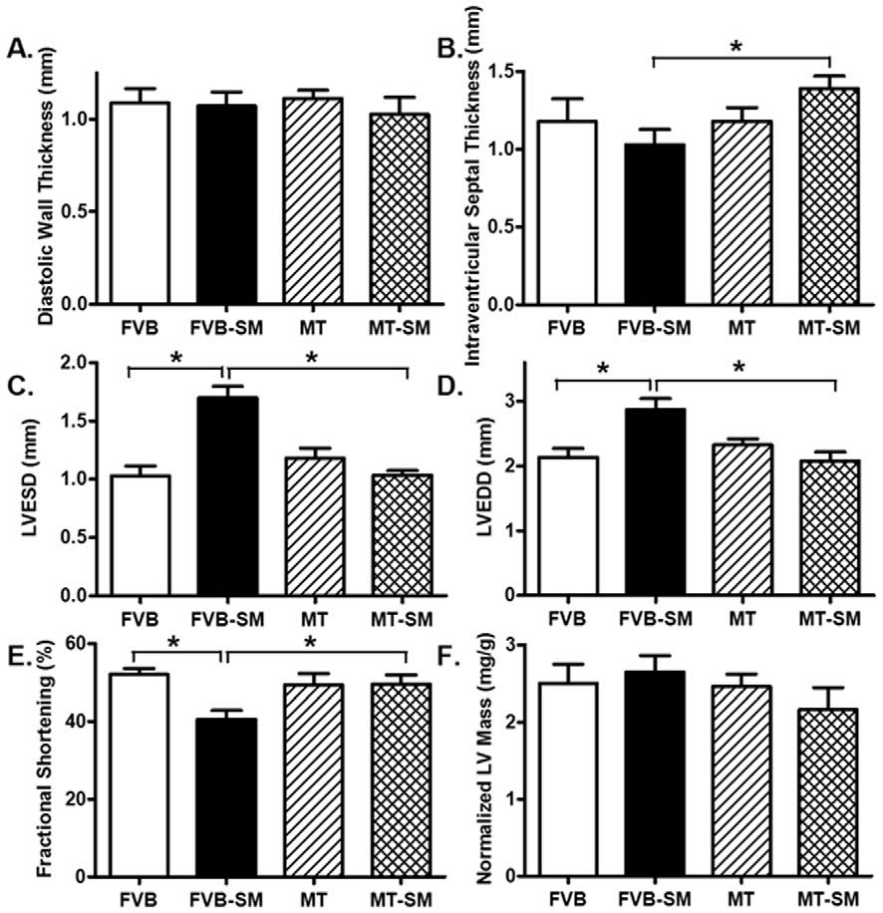
Effect of side-stream smoking (SM) on cardiac geometry and function using the M-mode echocardiography in FVB and metallothionein (MT) mice. A: Left ventricular (LV) diastolic wall thickness; B: Intraventricular septal thickness; C: LV end systolic diameter (LVESD); D: LV end diastolic diameter (LVEDD); E: Fractional shortening; and F: LV mass (normalized to body mass). Mean ± SEM, n = 7 mice per group, *p<0.05.

Neither side-stream smoke nor metallothionein overexpression, or both, affected heart rate, body, heart, liver, kidney weights and the heart size (heart-to-body weight ratio) as well as systolic and diastolic blood pressures. Side-stream smoke exposure significantly elevated plasma levels of proinflammatory cytokines including TNF-α and IL-6 as well as the circulating marker for systematic oxidative stress 8-isoprostane without affecting nitric oxide (NOx) levels in both FVB and metallothionein transgenic mice. Metallothionein transgene itself did not affect these plasma biochemical markers. Neither side-stream smoke nor metallothionein, or both, affected plasma levels of glucose or insulin. Side-stream smoke exposure significantly reduced GSH levels and GSH/GSSG ratio without altering GSSG levels in hearts, the effect of which was alleviated by metallothionein. Metallothionein itself did not elicit significant effect on myocardial levels of GSH, GSSG and GSH/GSSH ratio (Table 1). Echocardiographic test revealed comparable left ventricular wall thickness and LV mass among four mouse groups. However, side-stream smoke exposure significantly increased LV end systolic diameter (LVESD) and LV end diastolic diameter (LVEDD) as well as lessened fractional shortening in hearts from FVB mice, the effect of which was abrogated by metallothionein. Metallothionein itself did not affect LVESD, LVEDD and fractional shortening. In addition, side-stream smoke exposure significantly increased intraventricular septal thickness in metallothionein although not in FVB mice (Fig. 1).

**Table 1:** General biometric and biochemical characteristics of FVB and MT mice with or without side-stream smoke exposure (1 hour daily for 40 days).

		FVB	FVB-SM	MT	MT-SM
Biometric	Body Weight (g)	29.0±0.9	30.1±0.8	30.7±0.5	30.5±0.7
	Heart Weight (mg)	138±4	135±2	135±2	132±3
	Heart/Body Weight (mg/g)	4.80±0.18	4.50±0.12	4.42±0.08	4.35±0.14
	Liver Weight (g)	1.28±0.02	1.29±0.03	1.29±0.02	1.29±0.02
	Kidney Weight (g)	0.40±0.02	0.37±0.04	0.35±0.02	0.38±0.01
	Diastolic Blood Pressure (mmHg)	92±2	86±2	84±2	89±3
	Systolic Blood Pressure (mmHg)	112±2	111±2	104±0.02	108±2
	Mean Blood Pressure (mmHg)	94±2	92±11	88±2	91±2
	Heart rate (bpm)	690±19	708±9	700±16	710±11
Biochemical	Blood glucose (mg/dl)	103±3	99±4	101±4	101±6
	Plasma insulin (ng/ml)	0.369±0.037	0.412±0.045	0.355±0.034	0.400±0.033
	Plasma TNF-α (pg/ml)	0.440±0.031	0.878±0.045^*^	0.418±0.045	0.884±0.053^*^
	Plasma IL-6 (pg/ml)	0.652±0.036	0.894±0.068^*^	0.660±0.043	0.859±0.111^*^
	Plasma 8-isoprostane (pg/ml)	50±3	146±16^*^	50±6	160±21^*^
	Plasma nitric oxide (NOx, µM)	0.468±0.058	0.436±0.081	0.413±0.088	0.482±0.063
	Cardiac GSH (nmol/mg tissue)	116.8±7.5	85.5±9.8^*^	112.4±8.5	110.4±8.2
	Cardiac GSSG (nmol/mg tissue)	48.2±5.2	47.6±5.1	48.2±7.6	45.9±6.9
	Cardiac GSH/GSSG Ratio	2.42±0.27	1.79±0.25^*^	2.33±0.24	2.41±0.24

Mean ± SEM, n = 7–9 mice per group, ^*^ p<0.05 *vs*. FVB or MT group.

### Effect of side-stream smoke exposure on cardiomyocyte contractile and intracellular Ca^2+^ properties

**Figure 2:**
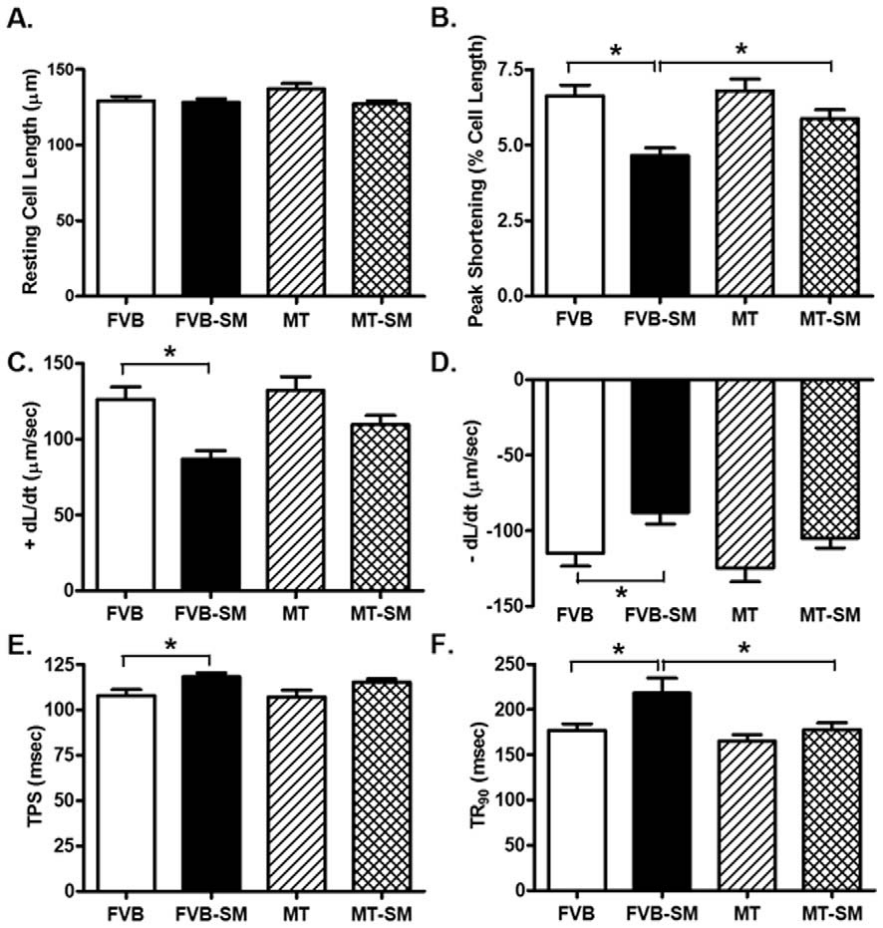
Effect of side-stream smoking (SM) on cardiomyocyte contractile properties in FVB and metallothionein (MT) mice. A: Resting cell length; B: Peak shortening (% of resting cell length); C: Maximal velocity of shortening (+ dL/dt); D: Maximal velocity of relengthening (− dL/dt); E: Time-to-peak shortening (TPS); and F: Time-to-90% relengthening (TR_90_). Mean ± SEM, n = 90–96 cells from 3 mice per group, * p<0.05.

**Figure 3:**
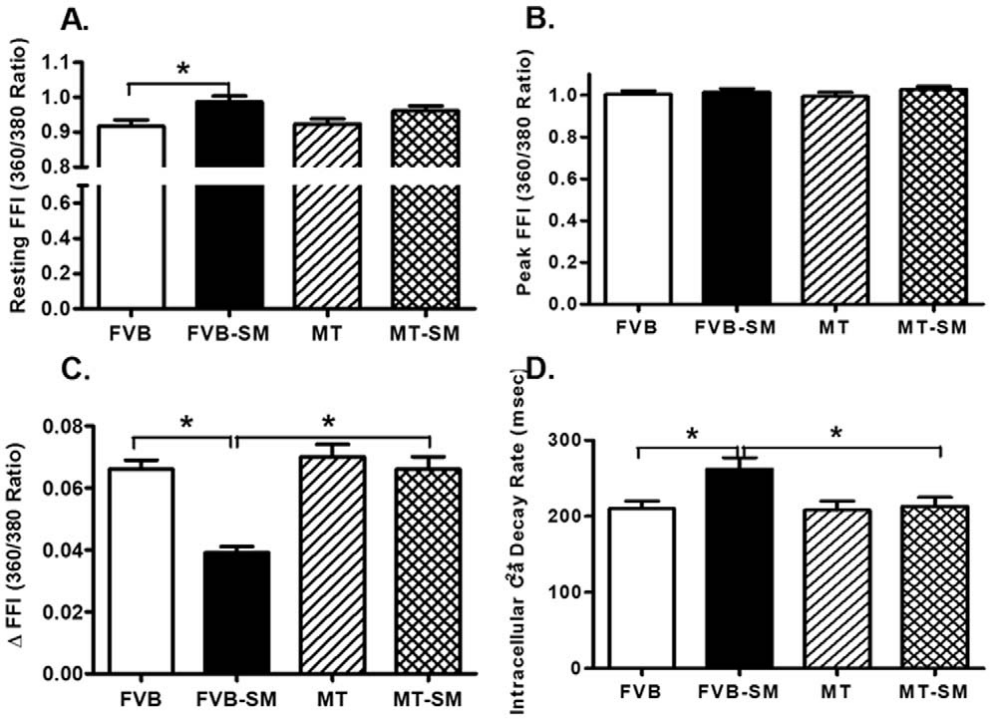
Effect of side-stream smoking (SM) on intracellular Ca^2+^ transients measured using Fura-2 in cardiomyocytes from FVB and metallothionein (MT) mice. A: Baseline fura-2 fluorescence intensity (FFI); B: Change in FFI (ΔFFI) in response to electrical stimuli; C: Single exponential intracellular Ca^2+^ decay rate; and D: Bi-exponential intracellular Ca^2+^ decay rate. Mean ± SEM, n = 71–77 cells from 3 mice per group, * p<0.05.

Neither short-term side-stream smoke exposure nor metallothionein overtly affected the phenotype (data not shown) or resting cell length in cardiomyocytes. Cardiomyocytes from side-stream smoke exposed FVB mice displayed significantly depressed PS and ± dL/dt as well as prolonged TPS and TR_90_. Although overexpression of metallothionein itself did not affect these mechanical parameters tested, it significantly attenuated or abrogated side-stream smoke exposure-induced mechanical changes (Fig. 2). To further understand the possible mechanism of action behind side-stream smoke exposure and metallothionein-induced myocardial mechanical responses, intracellular Ca^2+^ homeostasis was evaluated in cardiomyocytes using the intracellular Ca^2+^ fluorescent dye Fura-2. Data presented in Fig. 3 displayed that side-stream smoke exposure significantly elevated baseline fura-2 fluorescence intensity (FFI) and suppressed electrically-stimulated rise in fura-2 fluorescence intensity (ΔFFI) as well as slowed down intracellular Ca^2+^ decay rate without affecting the peak FFI. Although metallothionein itself did not affect these intracellular Ca^2+^ parameters, it abolished side-stream smoke exposure-induced changes in ΔFFI and intracellular Ca^2+^ decay rate without affecting the elevated baseline FFI. In addition, although neither side-stream smoke exposure nor metallothionein alone affected peak FFI, combination of the two significantly increased the peak FFI.

### Effect of side-stream smoke exposure on metallothionein expression, ROS production, aconitase activity and cytochrome c release

**Figure 4:**
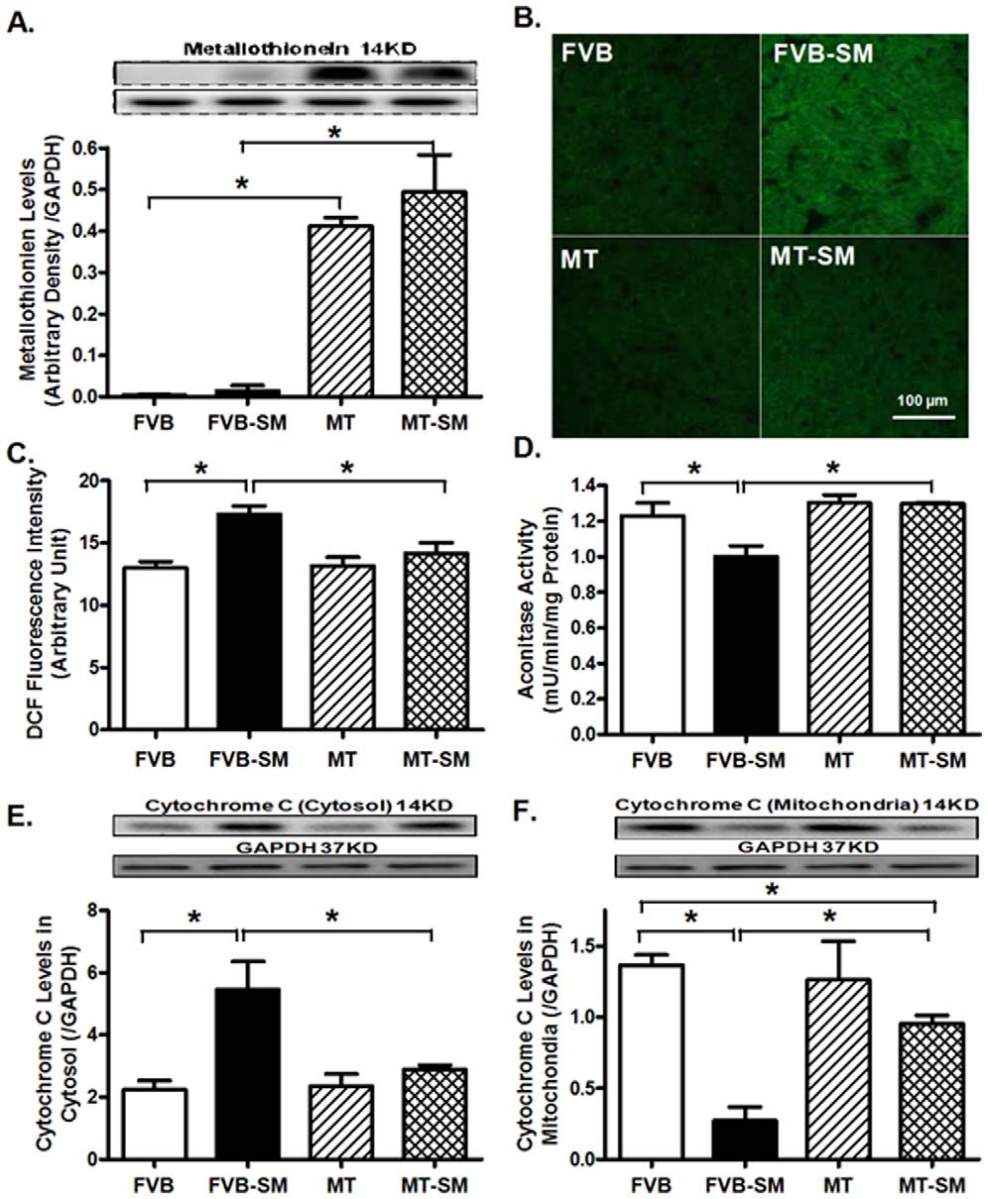
Effect of side-stream smoking (SM) on expression of metallothionein, ROS production, aconitase activity and cytochrome C distribution in FVB and metallothionein (MT) mice. A: Metallothionein; B: ROS production in myocardial sections using DCF green fluorescence; C: Pooled DCF fluorescence intensity; D: Aconitase activity; and E-F: Cytochrome c levels in the cytosol (E) and mitochondria (F). Myocardial tissues were separated using differential density centrifugation to yield cytosolic and mitochondrial fractions prior to gel electrophoresis. Inset: Representative gel blots depicting expression of metallothionein, cytochrome C and GAPDH (loading control) using specific antibodies. Mean ± SEM, n = 3–4 mice (or 11–14 slices) per group; * p<0.05.

Our data shown in Fig. 4A confirmed overtly higher metallothionein protein levels in the metallothionein transgenic mice, validating the transgenic model. Side-stream smoke exposure did not affect metallothionein expression. To assess the effect of side-stream smoke exposure and/or metallothionein on ROS generation, left ventricular tissues from FVB and metallothionein mice with or without side-stream smoke exposure were stained with DCF fluorescence dye prior to assessment of ROS. Our data revealed that smoke exposure overtly enhanced ROS generation, the effect of which was mitigated by metallothionein, in a manner reminiscent of myocardial contractile and intracellular Ca^2+^ responses. Metallothionein itself did not significantly affect ROS production (Fig. 4B-C). Our result further depicted that smoke exposure led to significantly higher cytosolic cytochrome C in conjunction with decreased mitochondrial cytochrome C levels, suggesting mitochondrial cytochrome C release. This is associated with significantly decreased aconitase activity following side-stream smoke exposure. Aconitase is an iron sulfur enzyme located in citric acid cycle, and the mitochondrial aconitase activity is closely associated with oxidative stress 21]. While metallothionein did not affect cytochrome C distribution or aconitase activity, it rectified side-stream smoke exposure-induced decrease in aconitase activity and mitochondrial cytochrome C release (Fig. 4D-F).

### Effects of side-stream smoke exposure on myocardial histology

**Figure 5:**
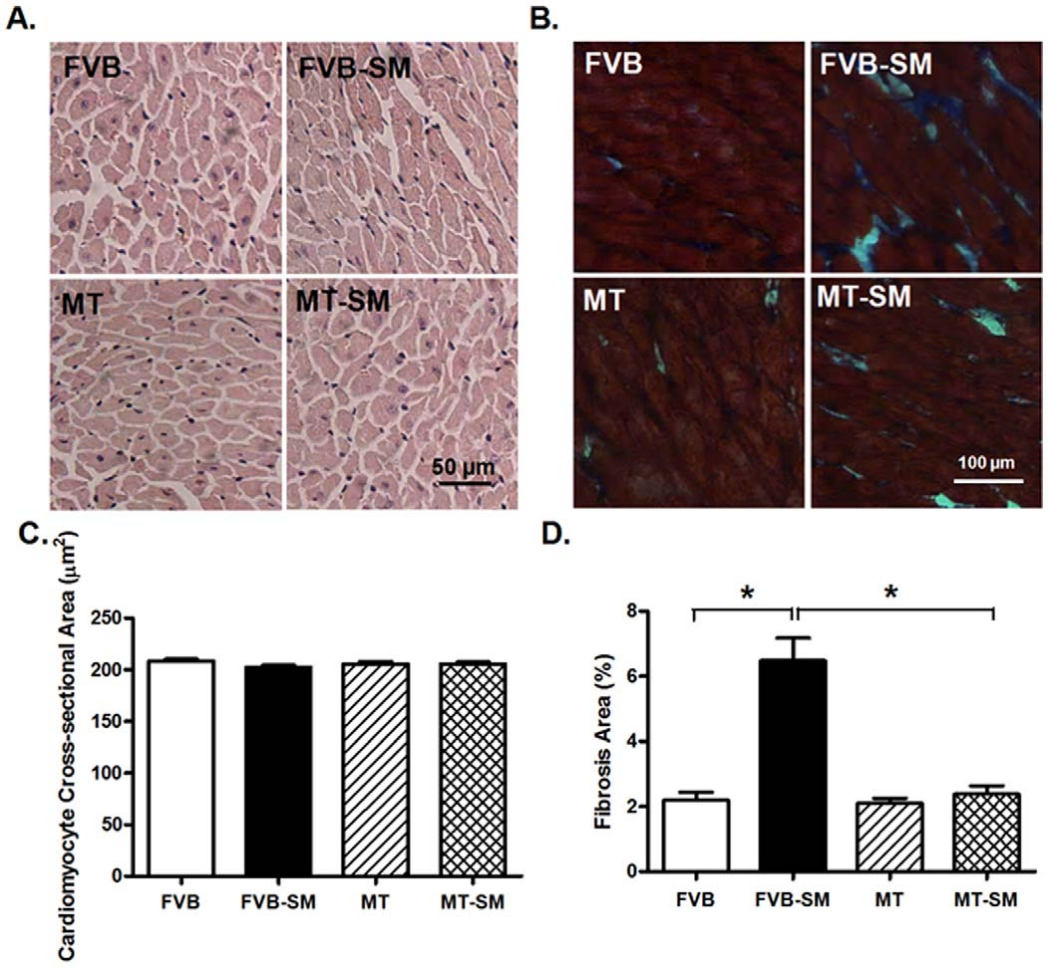
H&E and Masson's trichrome stained photomicrographs exhibiting cardiomyocyte cross-sectional area in FVB and metallothionein (MT) mice with or without side-stream smoking (SM) exposure. Panel A: Representative H&E staining in FVB and MT mice with or without smoke exposure; panel B: Masson trichrome staining exhibiting interstitial fibrosis; Panel C: Pooled data of cardiomyocyte cross-sectional area. Mean ± SEM, n = 200 cells from 10–15 fields of three mice per group; and panel D: Pooled data of myocardial fibrosis. Mean ± SEM, n = 10–15 fields from three mice per group, * p<0.05.

To assess the impact of side-stream smoke exposure and metallothionein on myocardial morphology, myocardial histology was assessed using H&E staining. Neither side-stream smoke nor metallothionein, or both, affected cardiomyocyte transverse cross-sectional area (Fig. 5A&C). Our study using the Masson trichrome staining revealed overt myocardial fibrosis following side-stream smoke exposure, the effect of which was ablated by the metallothionein transgene (Fig. 5B&D).

### Effect of side-stream smoke exposure on SERCA2a, PLB, eNOS expression

**Figure 6:**
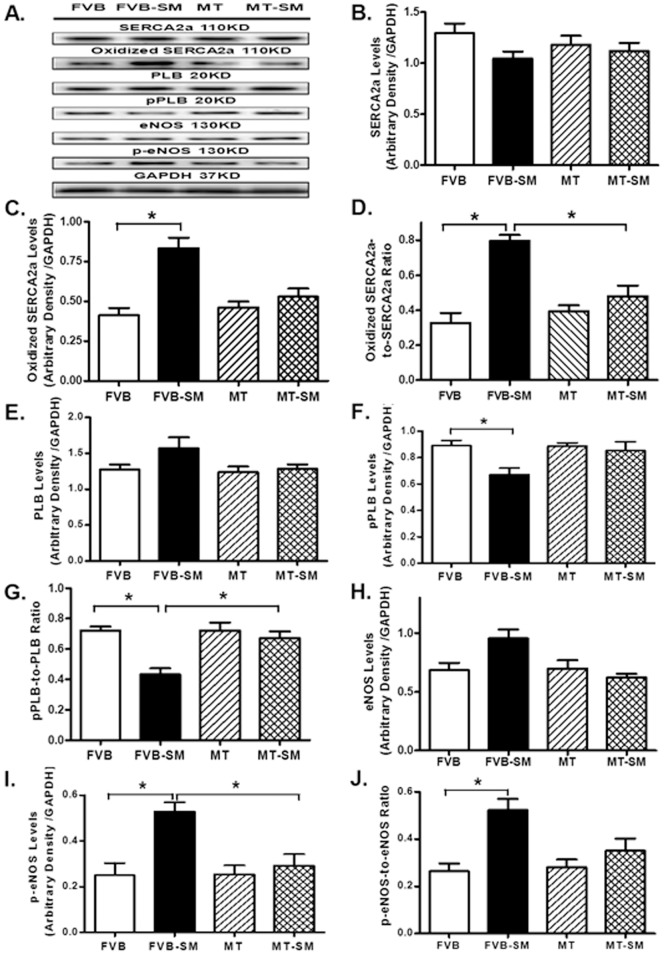
Effect of side-stream smoking (SM) on total and oxidized SERCA2a, total and phosphorylated phospholamban (PLB) and eNOS in myocardium from FVB and metallothionein (MT) mice. A: Representative gel blots depicting expression of SERCA2a, PLB, pPLB, eNOS and peNOS using specific antibodies. GAPDH was used as loading control. B: Total SERCA2a; C: Oxidative modification of SERCA2a detected by immunoprecipitation; D: Oxidative SERCA2a-to-total SERCA2a ratio; E: Total PLB; F: Phospho-PLB; G: pPLB-to-PLB ratio; H: total eNOS; I: Phospho-eNOS; J: peNOS-to-eNOS ratio. Mean ± SEM, n = 3–5 mice per group; * p<0.05.

To better understand the mechanism involved in smoke exposure and metallothionein-induced changes intracellular Ca^2+^ homeostasis, intracellular Ca^2+^ regulatory proteins SERCA2a and the SERCA inhibitory protein phospholamban as well as eNOS were evaluated. Our results revealed that while side-stream smoke exposure did not affect protein expression of SERCA2a, phospholamban and eNOS, it significantly dampened phosphorylation of phospholamban and promoted oxidation of SERCA2a and phosphorylation of eNOS (absolute or normalized value). Although metallothionein transgene did not affect total protein expression and phosphorylation (or oxidation) of SERCA2a, phospholamban and eNOS, it significantly attenuated or mitigated smoke exposure-elicited changes in the oxidation of SERCA2a, as well as phosphorylation of phospholamban and eNOS (Fig. 6).

### Effect of side-stream smoke exposure on total and phosphorylated Akt and GSK3β in FVB and metallothionein mice

**Figure 7:**
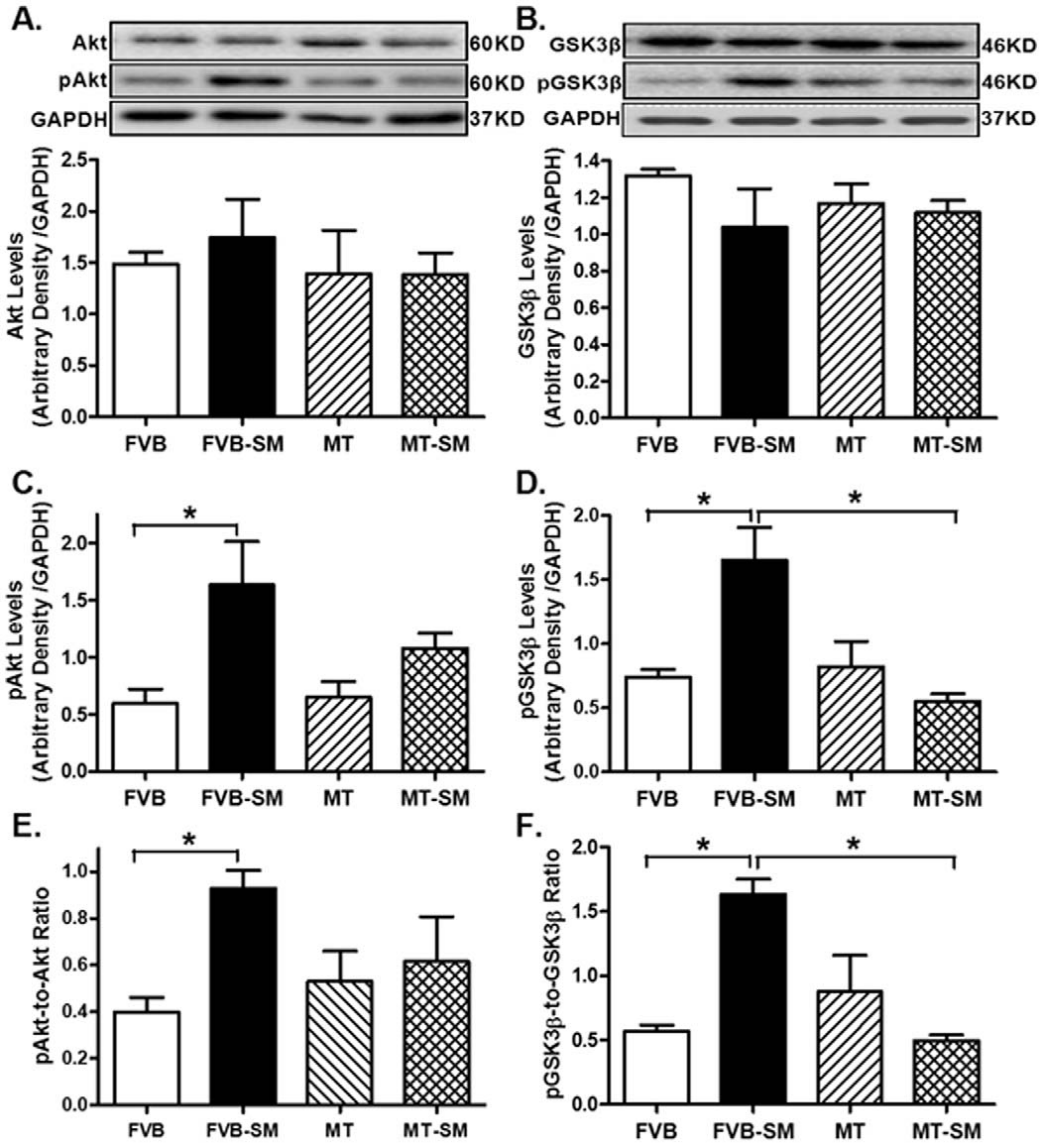
Effect of side-stream smoking (SM) on basal and phosphorylation levels of Akt and GSK3β in myocardium from FVB and metallothionein (MT) mice. A: Pan Akt; B: Pan GSK3β; C: Phospho-Akt (pAkt); D: Phospho-GSK3β; E: pAkt-to-Akt ratio; and F: pGSK3β-to-GSK3β ratio. Insets: Representative gel blots depicting expression of pan and phosphorylated Akt and GSK3β using specific antibodies. GAPDH was used as the loading control. Mean ± SEM, n = 3–5 mice per group; * p<0.05.

Our results indicated that while side-stream smoke exposure failed to alter total protein expression of Akt and GSK3β, it significantly increased the basal phosphorylation of Akt and GSK3β (absolute or normalized value). Although metallothionein failed to alter expression of total and phosphorylated Akt and GSK3β, it significantly attenuated or ablated side-stream smoke exposure-elicited increase in the phosphorylation of Akt and GSK3β (Fig. 7).

### Effect of side-stream smoke exposure on apoptotic protein makers

**Figure 8:**
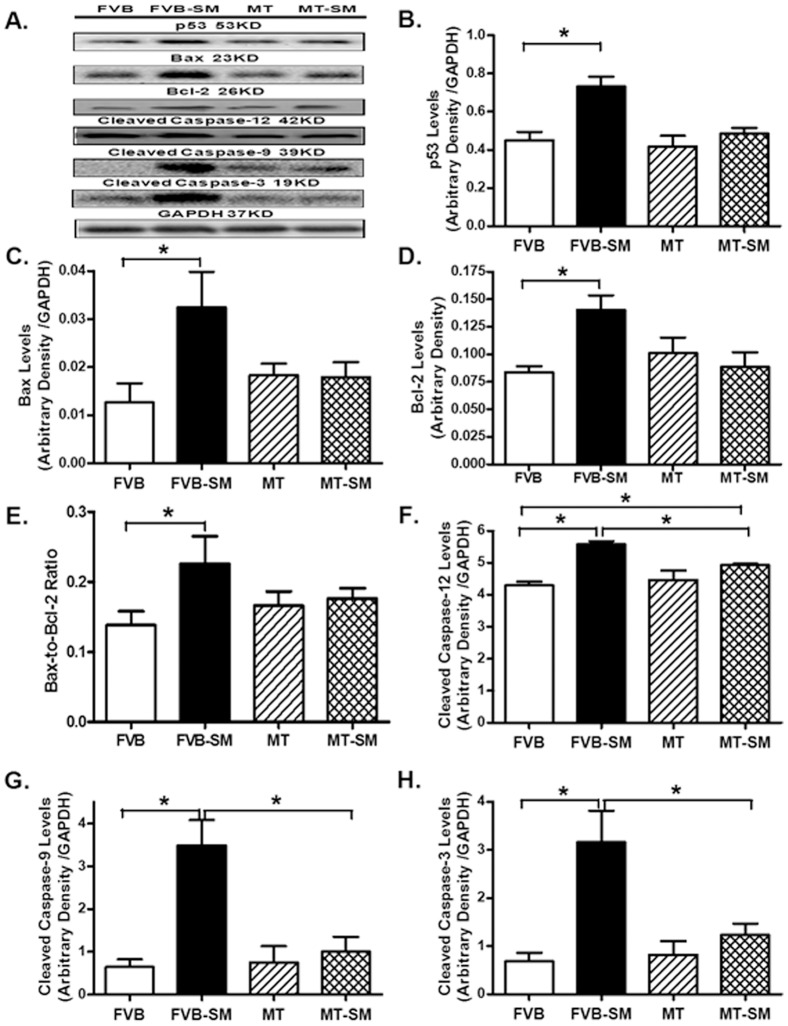
Effect of side-stream smoking (SM) expression of apoptotic proteins Bax, p53, Bcl-2, and caspase-3, -9 and -12 in FVB and metallothionein (MT) mice. A: Representative gel blots depicting expression of p53, Bax, Bcl-2, cleaved caspase-3, -9 and -12 using respective specific antibodies. GAPDH was used as loading control. B: Metallothionein expression; C: Bax expression; D: Bcl-2; E: Bax-to-Bcl-2 ratio; F: Cleaved Caspase-3 expression; G: Cleaved caspase-9 expression; and H: Cleaved Caspase-12 expression. Mean ± SEM, n = 4–6 per group; * p<0.05.

Our results shown in Fig. 8 further indicated that smoke exposure significantly upregulated the expression of the pro-apoptotic proteins Bax, p53, the anti-apoptotic protein Bcl-2, Bax-to-Bcl-2 ratio, caspase-3, caspase-9 and the ER-specific caspase-12. Although metallothionein itself failed to affect the expression of these apoptotic proteins, it significantly attenuated or mitigated smoke exposure-elicited responses in Bax, Bcl-2, Bax-to-Bcl-2 ratio, caspase-3, -9 and -12.

### Effect of SSE on cardiomyocyte contractile and intracellular Ca^2+^ properties

**Figure 9:**
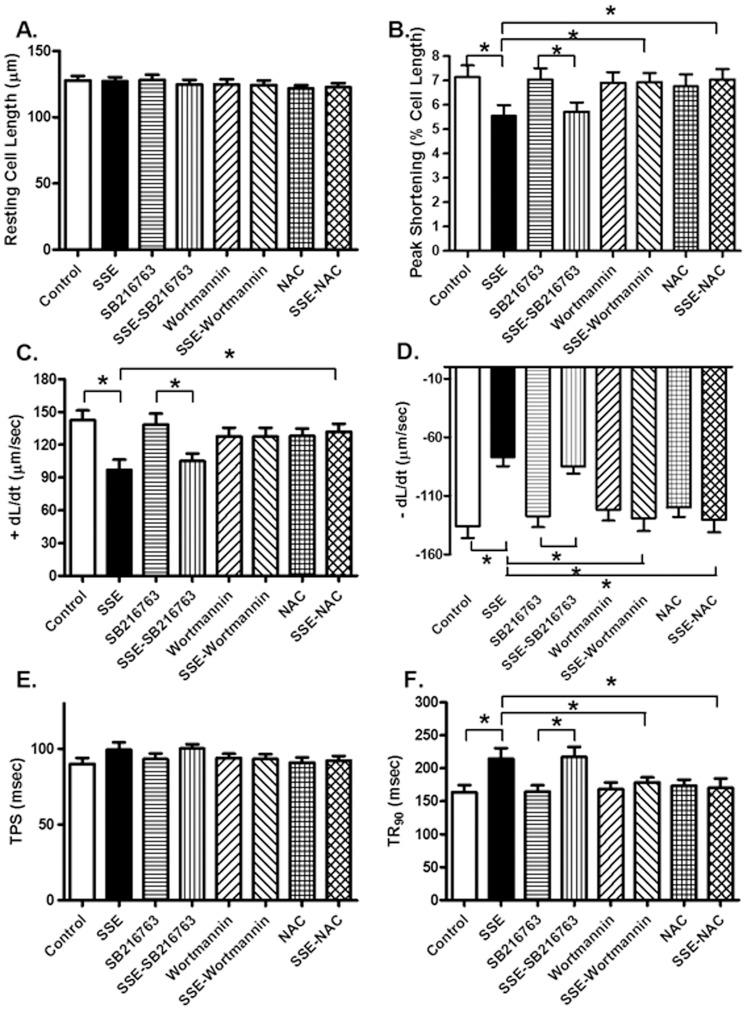
Effect of side-stream smoke extract (SSE) on *in vitro* cardiomyocyte contractile properties. Cardiomyocytes from FVB control mice were pretreated with the PI3K inhibitor wortmannin (100 nM), the GSK3β inhibitor SB216763 (100 nM) or the antioxidant N-acetylcysteine (NAC, 500 µM) for 1 h prior to exposure of 5% SSE (90 µg/ml) for 1 hr (with all inhibitors or NAC present). A: Resting cell length; B: Peak shortening (normalized to resting cell length); C: Maximal velocity of shortening (+ dL/dt); D: Maximal velocity of relengthening (− dL/dt); E: Time-to-peak shortening (TPS); and F: Time-to-90% relengthening (TR_90_). Mean ± SEM, n = 55–60 cells from 3 mice per group, * p<0.05.

**Figure 10:**
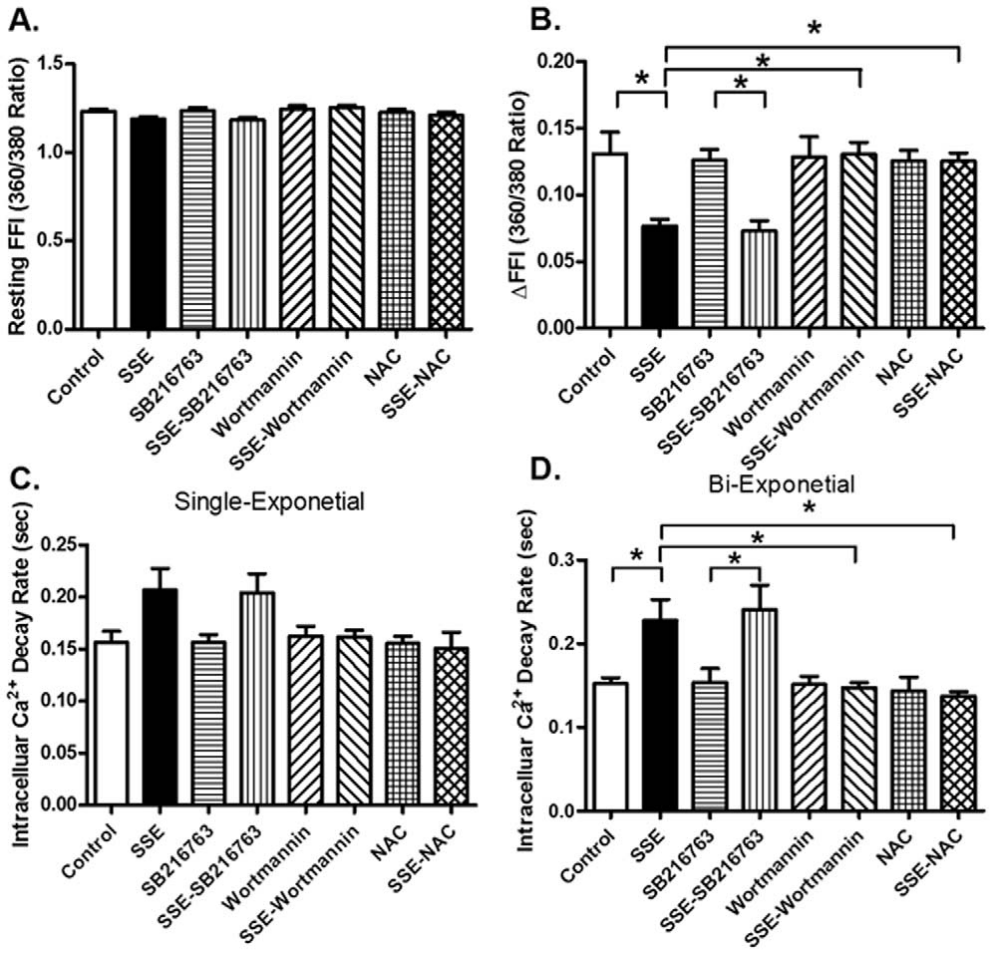
Effect of side-stream smoke extract (SSE) on *in vitro* cardiomyocyte intracellular Ca^2+^ transients. Cardiomyocytes from FVB control mice were pretreated with the PI3K inhibitor wortmannin (100 nM), the GSK3β inhibitor SB216763 (100 nM) or the antioxidant N-acetylcysteine (NAC, 500 µM) for 1 h prior to exposure of 5% SSE (90 µg/ml) for 1 hr (with all inhibitors or NAC present). A: Baseline fura-2 fluorescence intensity (FFI); B: Change in FFI (ΔFFI) in response to electrical stimuli; C: Single exponential fluorescence decay rate; and D: Bi-exponential fluorescence decay rate. Mean ± SEM, n = 55–60 cells from 3 mice per group, * p<0.05.

We further examined the effect of the smoke extract SSE on cardiomyocyte function *in vitro* in the absence or presence of the antioxidant N-acetylcysteine (NAC, 500 µM), inhibitors for PI3K wortmannin (100 nM) and GSK3β SB216763 (100 nM). Cardiomyocytes from FVB mice were pretreated with NAC, wortmannin or SB216763 for 1 hr prior to exposure of SSE (90 µg/ml). Resting cell length was not affected by SSE, NAC, wortmannin, or SB216763. Similar to its effects *in vivo*, 5% SSE exposure significantly decreased peak shortening, maximal velocity of shortening/relengthening (± dL/dt) and prolonged duration of relengthening (TR_90_) without affecting duration of shortening (TPS). Although wortmannin and NAC did not affect any of the mechanical indices tested, they abolished SSE-induced alterations in peak shortening, ± dL/dt and TR_90_. The GSK3β inhibitor SB216763 did not affect cardiomyocyte contractile properties in the absence or presence of SSE exposure (Fig. 9). We went on to examine the effect of the smoke extract SSE on intracellular Ca^2+^ handling in cardiomyocytes using the Fura-2 technique. Our data shown in Fig. 10 revealed that SSE exposure significantly inhibited electrically-stimulated rise in Fura-2 fluorescence intensity (ΔFFI) and prolonged intracellular Ca^2+^ clearance rate. Although the PI3K inhibitor wortmannin or NAC failed to alter intracellular Ca^2+^ properties, they effectively abolished the SSE-induced alterations in intracellular Ca^2+^ mishandling (reduced ΔFFI and prolonged intracellular Ca^2+^ clearance). Reminiscent of its action on cardiomyocyte contraction, the GSK3β inhibitor SB216763 did not intracellular Ca^2+^ homeostasis the absence or presence of SSE exposure (Fig. 10).

## Discussion

The major findings from our study revealed that short-term side-stream smoke exposure elicits overt cardiac oxidative stress, interstitial fibrosis, geometric, contractile and intracellular Ca^2+^ dysfunction (evidenced by enlarged LVESD and LVEDD although not cardiomyocyte size, reduced fractional shortening, peak shortening, maximal velocity of shortening/relengthening, prolonged duration of shortening and relengthening, increased basal intracellular Ca^2+^ levels and reduced intracellular Ca^2+^ release in response to electrical stimuli). Intriguingly, cigarette smoke exposure-associated abnormalities in ROS accumulation, oxidative stress, interstitial fibrosis, and cardiac mechanical function were significantly attenuated or nullified by the heavy metal scavenger metallothionein. Furthermore, compromised myocardial function, intracellular Ca^2+^ handling and fibrosis following second-hand smoke exposure were accompanied with apoptosis (p35, Bax, Bcl-2, Bax-to-Bcl-2 ratio, Caspase-3, 9 and -12) and mitochondrial damage (reduced aconitase activity and elevated cytochrome C release from mitochondria). *In vitro* examination using the antioxidant NAC and PI-3 kinase-Akt inhibitor revealed that smoke extract may impair cardiomyocyte contractile function, consistent with the *in vivo* findings of enhanced Akt phosphorylation and oxidative stress in the heart. These findings have collectively suggested a favorable role of antioxidants in second-hand smoking-induced myocardial anomalies.

Cigarette smoking has been reported to compromise cardiac performance independent of coronary atherosclerosis, elevation of blood pressure and endothelial dysfunction 7]. This is supported by our present findings where echocardiographic fractional shortening, cardiomyocyte contractile capacity as well as duration of contraction and relaxation are impaired following side-stream smoke exposure. The unaffected levels of blood glucose, plasma insulin and blood pressure following side-stream smoke exposure does not favor a major contribution of diabetes mellitus, insulin resistance and hypertension to side-stream smoke exposure-induced myocardial aberrations in our current experimental setting, although one can argue that the relatively short duration of smoke exposure may play a role. This is supported by the unchanged plasma NOx levels following side-stream smoke exposure. Passive smoking has been shown to compromise cardiac mechanical function, leading to unfavorable cardiac remodeling 7,30]. Greater LV mass was reported in chronic smokers in the absence of changes in blood pressure and LV fractional shortening 31]. In a recent study, 32 weeks of cigarette smoke exposure triggered an increase in LV wall thickness with a decrease in stroke volume and cardiac output albeit unchanged global *in vivo* cardiac contractile function 7]. In our hands, we failed to note any significant change in LV mass (unchanged cardiomyocyte size from H&E staining analysis) despite apparent cardiac remodeling (increased LVESD and LVEDD) following short-term side-stream smoke exposure, suggesting chamber dilation in the absence of ventricular wall thickening (coinciding with unchanged wall thickness and cardiomyocyte cross-sectional area) following side-stream smoke exposure. In addition, we noted decreased fractional shortening and cardiomyocyte contractile capacity (decreased peak shortening and maximal velocity of shortening/relengthening) after 40 days of side-stream smoke exposure. Although it is still unclear with regards to the discrepancy between our current findings and those reported earlier 7,31], difference in the passive smoke exposure duration (40 days in our study versus the much longer exposure in the above mentioned study) and the physiological parameters measured (factional shortening in our study versus ejection fraction) may possibly play a role. Our data revealed prolonged duration of shortening and relengthening, impaired intracellular Ca^2+^ homeostasis (elevated baseline FFI, reduced ΔFFI and prolonged intracellular Ca^2+^ decay) along with overt SERCA2a oxidization and lessened phospholamban phosphorylation in hearts following side-stream smoke exposure. These findings indicate a pivotal role of intracellular Ca^2+^ mishandling in side-stream smoke exposure-induced myocardial dysfunction, consistent with the earlier findings that carbon monoxide from heavy smokers significantly lowered intracellular Ca^2+^ amplitude, prolonged intracellular Ca^2+^ decay, reduced capacity of SERCA2a, and lessened phosphorylation of phospholamban 32].

**Figure 11:**
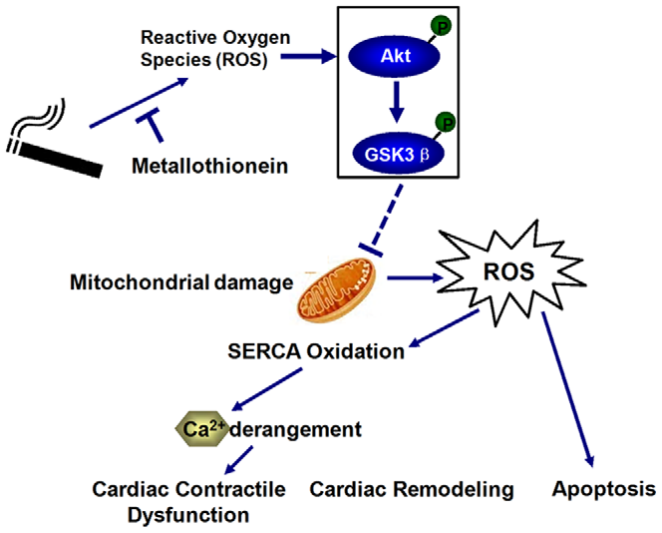
Schematic diagram depicting proposed signaling events in side-stream smoking- and metallothionein-induced cardiac responses. Dashed lines denote event not fully validated by this study.

Perhaps the most interesting finding from our study is that antioxidant metallothionein ablated or attenuated smoke exposure-induced myocardial defects, intracellular Ca^2+^ mishandling, cardiac remodeling (fibrosis), apoptosis and mitochondrial damage. This received further support from our *in vitro* finding that the antioxidant NAC negates smoke extract-induced cardiomyocyte contractile anomalies. Our data revealed that cardiac overexpression of metallothionein failed to affect smoke exposure-induced systemic pro-inflammatory and pro-oxidant changes in TNF-α, IL-6, and 8-isoprostane, suggesting relatively trivial role of systemic effect in the antioxidant-elicited cardioprotection. Given that oxidative damage may lead to cardiac intracellular Ca^2+^ mishandling 13,18], the beneficial effect of metallothionein on intracellular Ca^2+^ homeostasis following side-stream smoke exposure may be mediated via its potent antioxidant property, as reported elsewhere 33,34]. This is supported by metallothionein-elicited protection against smoke exposure-induced ROS accumulation and SERCA2a oxidization. Nonetheless, possible contribution from other antioxidant systems following smoke exposure should not be discounted. Antioxidants have shown a beneficial role against ischemia-induced neovascularization in mice exposed to cigarette smoke 8] although little information for the heart. Our data of elevated cytochrome c release and the reduced aconitase levels in hearts from side-stream smoke exposed mice support a role of mitochondrial damage in smoke exposure-triggered cardiac anomalies. This is in line with the finding that smoking promotes mitochondria injury via nicotine production 35,36]. Cytochrome c is an essential component of the electron transport chain in mitochondria 37]. It is likely that ROS-triggered mitochondrial injury may be responsible for myocardial mechanical dysfunction, and ultimately cardiac insufficiency following prolonged cigarette smoking 38], as summarized in Fig. 11. Smoke exposure is known to promote cardiomyocyte apoptosis through oxidative stress and disruption of apoptosis-related gene expression 10,39]. Our results revealed that metallothionein exerts protective effects against side-stream smoke exposure-induced apoptosis, in a manner similar to that of mechanical and intracellular Ca^2+^ responses, favoring a role of lessened apoptosis in metallothionein-induced beneficial effect against side-stream smoke exposure. Cytochrome c is also an intermediate in apoptosis, a controlled form of cell death used to kill cells in the process of development or in response to infection or DNA damage 40]. Cytochrome c is released by the mitochondria in response to pro-apoptotic stimuli 41]. This release of cytochrome c in turn activates Caspase-9, and subsequently Caspase-3, which are responsible for the ultimate cell death 42]. In this study, our current finding in the expression of Caspase-3, -9 and -12 supports a role of apoptosis in side-stream smoking- and metallothionein-induced cardiac mechanical responses. The elevated levels of Bcl-2, an anti-apoptotic protein, in response to side-stream smoke exposure may be compensatory in nature due to higher oxidative stress (as evidenced by reduced GSH-to-GSSG ratio. Last but not the least, our data failed to reveal that side-stream smoke exposure altered cardiac metallothionein levels, thus not favoring a role of reduced intrinsic cardiac antioxidant capacity in short-term smoke exposure-induced cardiac pathological changes.

One interesting finding from our current study was that side-stream smoke exposure led to activation of Akt and GSK3β in myocardium, consistent with the findings of activation of Akt and GSK3β in epithelial cell systems prompting development of lung cancer. It is believed that activation of PI3K signaling turns on Akt, resulting in inactivation of GSK3β via Akt-mediated phosphorylation 43]. Our results are also in line with the notion of a dose- and time-dependent activation of Akt in response to nicotine or NNK in lung cancer cells 44]. Phosphorylation of Akt by smoke exposure may govern many cellular responses including survival and contractile functions. Activated Akt should phosphorylate GSK3β at serine residue 9 to inactivate the kinase 45], thus leading to preserved mitochondrial integrity and cell survival. Although the precise mechanism underscoring the mismatch between Akt-GSK3β and mitochondrial function in our study still remains elusive, it is plausible to speculate that chronic Akt activation may facilitate maladaptive or deleterious consequences in the heart 46,47]. This is supported by our *in vitro* observation that PI-3 kinase inhibition using wortmannin prevented smoke extract-induced cardiomyocyte contractile anomalies. A scheme (Fig. 11) is provided to summarize likelihood mechanism of action underscoring side-stream smoke exposure- and metallothionein-induced cardiac responses.

Although our study suggested a possible role of oxidative stress and inflammation (TNF-α, IL-6, 8-isoprostane) in side-stream smoke exposure-induced cardiac contractile dysfunction, consistent with previous reports 48,49], other systematic factors should not be excluded at this time. For example, smoke exposure may be associated with low steroid hormone levels, which may be instrumental in explaining some adverse effects of tobacco smoke on female health and fertility 50]. Further study is warranted to better elucidate the cardiac versus non-cardiac origin of second-hand smoke exposure-induced heart diseases. It should be pointed out that one major limitation of our study was the lack of blood smoke (or particulate matter) levels in these side-stream smoke exposed mice due to technical difficulties. Having these blood values should help to exclude potential effects of metallothionein on smoke particulate matter diffusion in the body.

Both epidemiological and experimental evidence support the assertion that cigarette smoking increases the incidence of cardiac event 2]. Our finding of cardioprotective effect of the heavy metal scavenger metallothionein against side-stream smoke exposure-induced cardiac geometric, contractile, and intracellular Ca^2+^ anomalies depict a favorable role of antioxidants against cigarette smoking-induced cardiovascular diseases. Although our data seem to indicate a role of apoptosis and mitochondrial damage in second-hand smoking-induced cardiac defect, in-depth mechanistic inside remains to be elucidated. In particular, the interplay between cigarette smoking and cell signaling machineries responsible for mitochondrial damage and apoptosis remains to be determined. These approaches should help us to better understand the therapeutic value of antioxidants in the management of cigarette smoking-induced cardiovascular diseases.
